# Cartilage Turnover Reflected by Metabolic Processing of Type II Collagen: A Novel Marker of Anabolic Function in Chondrocytes

**DOI:** 10.3390/ijms151018789

**Published:** 2014-10-17

**Authors:** Natasja Stæhr Gudmann, Jianxia Wang, Sabine Hoielt, Pingping Chen, Anne Sofie Siebuhr, Yi He, Thorbjørn G. Christiansen, Morten Asser Karsdal, Anne Christine Bay-Jensen

**Affiliations:** 1 Nordic Bioscience A/S, Herlev Hovedgade 207, DK-2730 Herlev, Denmark; E-Mails: jxw@NordicBioscienceChina.com (J.W.); sho@nordicbioscience.com (S.H.); ppc@NordicBioscienceChina.com (P.C.); aso@nordicbioscience.com (A.S.S.) yhe@nordicbioscience.com (Y.H.); mk@nordicbioscience.com (M.A.K.); 2 Orthopedic Department Z, Gentofte University Hospital, Niels Andersensvej 65, DK-2900 Hellerup, Denmark; E-Mail: Thorbjoern.christiansen@regionh.dk

**Keywords:** cartilage, biomarkers, type II collagen, IGF-1, TGF-β1, chondrocytes

## Abstract

The aim of this study was to enable measurement of cartilage formation by a novel biomarker of type II collagen formation. The competitive enzyme-linked immunosorbent assay (ELISA) Pro-C2 was developed and characterized for assessment of the beta splice variant of type II procollagen (PIIBNP). This is expected to originate primarily from remodeling of hyaline cartilage. A mouse monoclonal antibody (Mab) was raised in mouse, targeting specifically PIIBNP (QDVRQPG) and used in development of the assay. The specificity, sensitivity, 4-parameter fit and stability of the assay were tested. Levels of PIIBNP were quantified in human serum (0.6–2.2 nM), human amniotic fluid (163–188 nM) and sera from different animal species, e.g., fetal bovine serum (851–901 nM) with general good linearity (100% (SD 7.6) recovery) and good intra- and inter-assay variation (CV% < 10). Dose (0.1 to 100 ng/mL) and time (7, 14 and 21 days) dependent release of PIIBNP were evaluated in the conditioned medium from bovine cartilage explants (BEX) and human cartilage explants (HEX) upon stimulation with insulin-like growth factor (IGF-1), transforming growth factor (TGF)-β1 and fibroblastic growth factor-2 (FGF-2). TGF-β1 and IGF-1 in concentrations of 10–100 ng/mL significantly (*p* < 0.05) induced release of PIIBNP in BEX compared to conditions without treatment (WO). In HEX, IGF-1 100 ng/mL was able to induce a significant increase of PIIBNP after one week compared to WO. FGF-2 did not induce a PIIBNP release in our models. To our knowledge this is the first assay, which is able to specifically evaluate PIIBNP excretion. The Pro-C2 assay seems to provide a promising and novel marker of type II collagen formation.

## 1. Introduction

Degenerative joint disease (DJD) also known as osteoarthritis (OA) is characterized by progressive damage to the joint tissue, including traumatic lesions, fissuring and denudation of the articular cartilage [1]. The extracellular matrix (ECM) of healthy cartilage is characterized by a low turnover of collagens, whereas the turnover rate of aggrecan is relatively high [2]. In diseased cartilage the homeostasis is disrupted leading to an imbalance between cartilage formation and degradation resulting in a net loss of cartilage [3]. There are several examples of disease modifying OA drugs (DMOADs) in development that aim to protect the ECM balance either by inhibiting cartilage degradation or inducing formation [4]. Investigation is therefore initiated for biomarkers to evaluate cartilage in DJD. Magnetic resonance imaging (MRI) gives a promising outcome for evaluation of knee OA [5]. However widespread use of MRI is limited due to cost and it has been questioned whether detected changes are clearly correlated to beneficial outcomes for the patient [6]. Thus there is a need for other markers in proof of concept studies as well as for clinical trials of novel DMOADs candidates.

Type II collagen is the main organic component of articular cartilage [7]. Other components include proteoglycans, aggrecans, collagen IX and XI [8]. During synthesis of type II collagen, procollagen is formed and released as triple helical structures of three α-chains. Each α-chain is characterized by attachment of propeptides at the termini. The propeptides found in the *N*-terminal and the *C*-terminal are termed PIINP and PIICP, respectively. Procollagen is converted to mature type II collagen when these propetides are cleaved of by specific proteinases (*N*-proteinase and *C*-proteinase). The type II collagen is then deposited into fibrils of the ECM [9]. PIINP exists in two splice variants termed PIIANP and PIIBNP. The expression of PIIANP is usually restricted to embryogenesis, but may be re-expressed in OA [10]. This splice variant is thought to represent a dedifferentiated version of type II collagen and it is characterized by a prolongation of 69 amino acids in a cysteine-rich globular domain compared to PIIBNP [11]. PIIBNP, on the other hand, is considered to be the splice variant that is mainly expressed during type II collagen formation in healthy adults [12].

Several biomarkers are available for monitoring cartilage degradation; however only few for assessment of cartilage formation. Both formation and degradation biomarkers are needed for assessing remodeling of cartilage as a part of disease or as a measure of treatment mode of action. For evaluation of cartilage turnover there are different serological biomarkers for evaluation of degradation available including a neoepitope CIIM [13], a *C*-terminal telopeptide CTX-II [14], an inter-helical fragment TIINE [15], a neoepitope CIINE [16] and a neoepitope C2C [17] for type II collagen and the neoepitope biomarkers AGNX-II [18] and AGG-C1 [19] for aggrecan together with the biomarker for cartilage oligomeric matrix protein COMP [20]. Most of these biomarkers are validated in terms of *ex vivo* models, in collagen-induced arthritis and in subjects with either OA or rheumatoid arthritis (RA). The field of anabolic biomarkers is however less systematically investigated. The different existing biomarker variants include total PIINP [21,22,23] and PIICP [24], which give estimates of the total type II collagen formation. In addition there is a biomarker for assessment of PIIANP [11], which is used as an anabolic marker for OA [25] and RA [11].

We hypothesized that PIIBNP is a relevant estimate of anabolic stimuli in cartilage as it is the splice variant associated with formation of healthy, adult cartilage. The existing number of cartilage formation markers is as mentioned limited, which make a potential need for such a biomarker in order to evaluate cartilage protective and anabolic effects of drug candidates *in vitro* as well as *in vivo*. We therefore aimed to develop a robust biomarker assay that enables quantification of cartilage formation.

## 2. Results

### 2.1. Specificity of the Monoclonal Antibody

**Figure 1:**
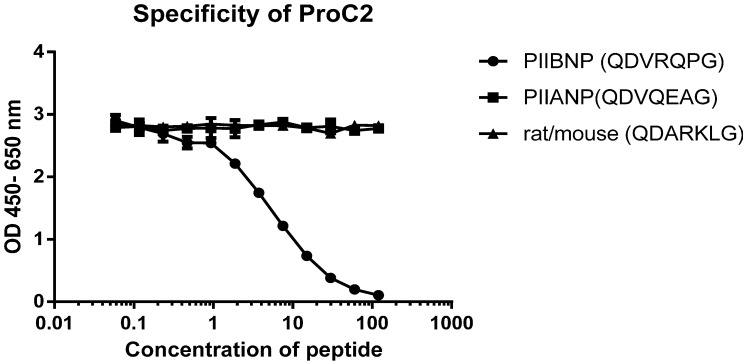
It was found that the NB443-3-2-1 antibody showed great potential for development of a competitive ELISA assay. The antibody was very specific towards peptides of the targeted PIIBNP sequence of which 120 ng/mL peptide was enough to displace the signal, whereas it did not recognize the PIIANP or the rat/mouse splice variant.

No cross reactivity was observed when specificity of the antibody was tested against peptides of the PIIANP and the corresponding rat/mouse isotype (see Experimental Section 4.1). This indicated that the developed antibody NB443-3-2-1 (see Experimental Section 4.2) was specific for the selected peptide as shown at Figure 1.

### 2.2. Technical Performance of the Pro-C2 ELISA

A technically robust competition assay was performed and is summarized in Table 1. The lower limit of detection (LLOD) was 0.96 nM. The measurement range was 2.5–19 nM and the IC_50_ was 7.82 nM. The intra-assay coefficient of variation (CV) was 10.8% and the inter-assay CV was 9.2%. The peptide spiking recovery test in human serum was 100% ± 20%, within the measurement range of the assay.

**Table 1:** Summary of the technical performance for Pro-C2.

Assay Specifications	Pro-C2
Slope of standard curve	1.03
IC50, nM	7.82
Intra-assay CV%	10.8
Inter-assay CV%	9.2
Lower limit of detection, nM	0.96
Quantifiable range, nM	2.5–19

### 2.3. PIIBNP Levels Measured in Serum of Humans and Other Species

Levels of PIIBNP were quantified in healthy adult humans, and turned out to be equal to or just above the LLOD. This made it difficult to give exact estimates. When the assay was applied in samples with high PIIBNP concentrations, such as fetal bovine serum (FBS) (851–901 nM) or amniotic fluid (163–187 nM), the assay showed excellent linearity (dilution range 1:2–1:64 and 1:20–1:160 respectively with 100% (SD 7.6) recovery).

### 2.4. PIIBNP Release in Bovine Cartilage Explants

**Figure 2:**
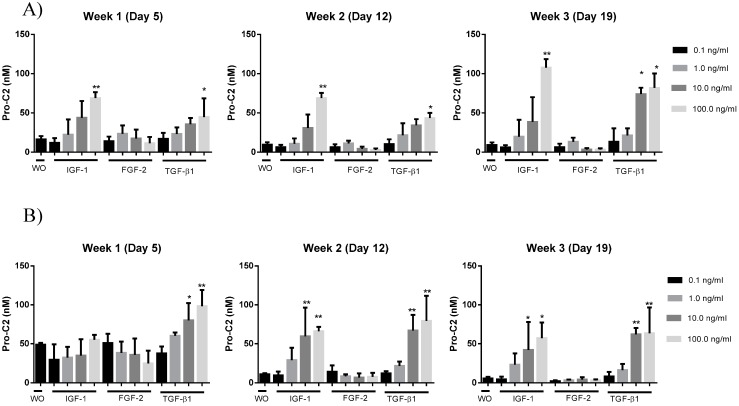
PIIBNP evaluated in supernatant of BEX cultured for 3 weeks in the presence 0.1–100 ng/mL of IGF-1, FGF-2 or TGF-β1. BEX cultured without treatment (WO) were applied for comparison of treatment effect. The experiment was performed twice, shown as (**A**) and (**B**) respectively to investigate if the results were reproducible. * *p* < 0.05, ** *p* < 0.005.

A bovine cartilage explants (BEX) model was useful to evaluate PIIBNP levels by mean of the developed Pro-C2 assay. Explants were cultured for 21 days with different anabolic cytokines in concentrations between 0.1 and 100 ng/mL in a 10 fold dilution range. It was found that transforming growth factor (TGF)-β1 and insulin-like growth factor (IGF)-1 significantly induced secretion of PIIBNP in a dose-dependent manner. This increased secretion was statistically significant from week 1 when 100 ng/mL was added compared to those without treatment (WO). At week 3 this difference between WO and IGF-1 was significant for a dose of 100 ng/mL and for both 10 and 100 ng/mL TGF-β1 compared to WO as shown at Figure 2A.

The same pattern was seen when the experiment was performed a second time with a knee from a different but age matched cow (Figure 2B). This time it was found that WO explants had a higher level of PIIBNP in week 1 (49 nM in average) compared to the first time the experiment was performed (16.1 nM in average). This time it was TGF-1β only, which was able to induce a significant PIIBNP response at week 1 compared to the WO. Similarly to the first experiment (Figure 2A) PIIBNP levels were significantly increased when stimulated by 100 ng/mL IGF-1 and TGF-1β in weeks 2 and 3 compared to the unstimulated samples. This time the levels of PIIBNP tended to decrease from 49 nM in week 1 to an average below 11 and 9 nM in weeks 2 and 3 respectively (Figure 2B). In comparison the levels of PIIBNP in unstimulated explants started at 16.1 nM at average in week 1 and decreased to below 10 in weeks 2 and 3 the first time the experiment was performed (Figure 2A).

**Figure 3:**
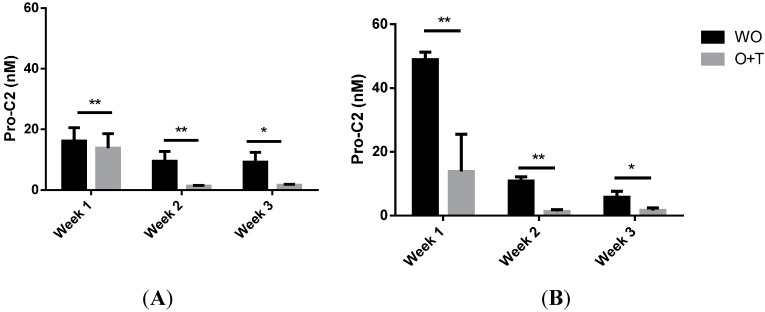
Pro-C2 measured in supernatant of BEX cultured for 3 weeks in presence of catabolic stimuli Oncostatin M + TNF-α (O+T) compared to unstimulated (WO) explants. The experiment was performed twice (**A**) and (**B**) respectively to investigate if the results were reproduceable. * *p* < 0.05, ** *p* < 0.005.

To investigate if PIIBNP secretion was affected by catabolic cytokines explants were treated by a combination of Oncostatin M + TNF-α (O+T) in parallel to the conditions of anabolic cytokines of both experiments (as seen in Figure 3). Both times it was found that the O+T treated explants had a significantly lowered PIIBNP secretion in weeks 2 and 3 compared to WO.

### 2.5. Size of the Detected Fragments in Supernatant

**Figure 4:**
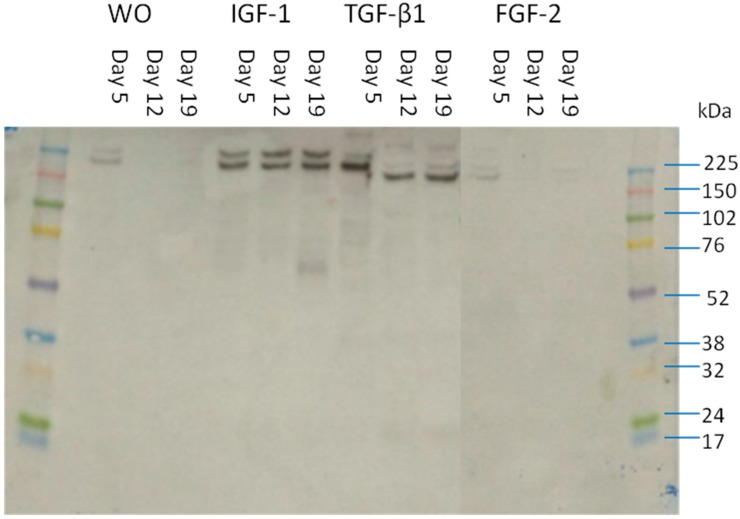
Western blot of the NB443-3-2-1 antibody detected fragments in supernatant of the BEX cultures.

Supernatants from BEX experiments were run through an SDS gel and the fragments were detected by the chosen antibody and evaluated by a western blot (Figure 4). The detected bands had a size of about 150 kDa, which corresponds to the size of alpha chains in type II collagen. Bands were barely detectable in supernatant from explants that were either not stimulated or stimulated by FGF-2, whereas bands were clearly seen in supernatant of BEX stimulated by either IGF-1 or TGF-1β.

### 2.6. PIIBNP Measured in Human Cartilage Explants

**Figure 5:**
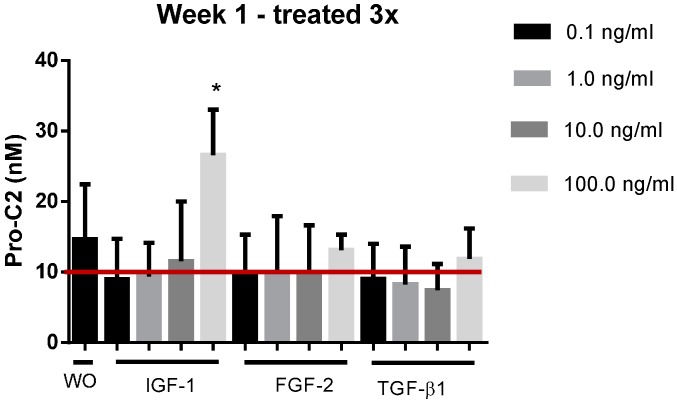
Pro-C2 evaluated in supernatant from HEX cultured in presence 0.1–100 ng/mL of IGF-1, FGF-2 or TGF-β1. HEX cultured without treatment (WO) was applied for comparison of treatment effect. The red line indicates the average background level of 10 nM originating from the FBS of the media. Error bars are shown as SEM. * *p* < 0.05.

Pro-C2 was applied to evaluate PIIBNP in human cartilage explants (HEX) from OA patients. In this model it was found that the measured levels were significantly increased at day 6 for explants stimulated by 100 ng/mL compared to WO as seen on Figure 5. The red line on the figure indicates the average background level originating from the addition of 0.5% FBS. Hence when background levels were subtracted the remaining PIIBNP measured in HEX were in general lower compared to those measured in BEX.

**Figure 6:**
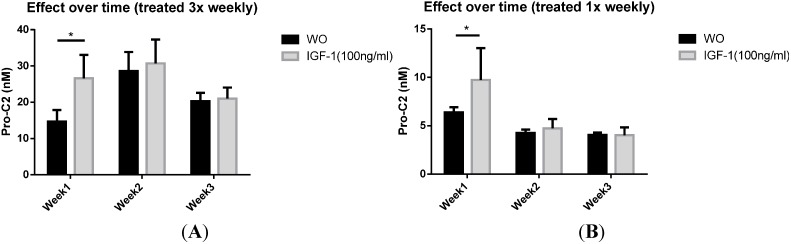
Pro-C2 evaluated in supernatant from HEX cultured with or without presence of 100 ng/mL IGF-1 for 3 weeks. (**A**) The PIIBNP concentration in supernatants from HEX stimulated by 100 ng/mL IGF-1 3 times per week; (**B**) The concentration in supernatants of HEX stimulated once a week. Error bars are shown as SEM. * *p* <0.05.

This increase of PIIBNP secretion seemed to disappear when HEX explants were cultured for 2 weeks or longer. This was tested in settings of which explants were exposed to a new dosage of IGF-1 either 3 times a week or in conditions of which IGF-1was added to the media once a week only, to ensure that the effect was not due to overstimulation (Figure 6).

## 3. Discussion

The ECM of cartilage constitutes a complex network of proteins of which type II collagen is the main component. In addition the expression of type II collagen is in adults found in hyaline cartilage, the vitreous body of eyes and the nucleus pulposus of intervertebral discs exclusively. Type II collagen has structural properties and keeps cartilage at homeostasis through interactions with polysaccharides and proteoglycans [26].

In OA, changes in cartilage start to occur as degradation exceeds formation. Anabolic treatment of articular cartilage is therefore a target for treatment, but it is currently difficult to evaluate the efficacy thereof. ProC-2 represents a novel biochemical marker reflecting cartilage formation. We have shown that the selected antigen is specific for the PIIBNP splice variant. That is what differentiates our biochemical marker from the related formation markers PIIANP [10] and F75 [22]. There may even be a need for the assay as a formation estimate combined with a marker of PIIANP in order to improve the accuracy of formation in the future as it was suggested by Garvican *et al.* [27]. A head to head comparison between our Pro-C2 marker and the PIIANP in its existing form is not possible though; as seen from Table 2, levels are quite low (0.6–2.2 nM) compared to the range of PIIANP which was evaluated, in a recently published study of healthy monozygotic and dizygotic twins, to be 650 ng/mL (CI 95% 623–677 ng/mL) and 624 ng/mL (CI 95% 599–649 ng/mL), respectively [28]. One might wonder if the measured PIIANP levels are unrealistically high for the origin of cartilage formation alone since type II collagen turnover in adults under normal circumstances is considered to be relatively low [2].

**Table 2:** Results of PIIBNP measured in sera from healthy adult humans and different animal species and human amniotic fluid. The concentrations are given as the average of the measured values.

Species	PIIBNP (nM)
Human (adult) serum	1.5
Amniotic fluid	175
Fetal bovine serum	875
Bovine serum	9
Fetal horse serum	415
Chicken serum	0.3
Rabbit serum	0.2

To our knowledge this is the first study that presents an assay specific for the PIIBNP splice variant. In our study: (I) The monoclonal antibody selected for the assay development was highly specific towards the selection peptide when tested in a competitive ELISA and there was no indication of cross-reactivity with the synthetically generated form of the epitope of PIIANP nor the mouse/rat sequence; (II) Due to sequence homology the Pro-C2 assay had a strong reactivity towards sera from different species and towards human amniotic fluid; (III) A technically robust assay was developed with acceptable inter-, and intra-assay variation, dilution and spiking recovery; (IV) In the *ex vivo* part of the study, PIIBNP levels were significantly higher in BEX explants stimulated by the anabolic cytokines IGF-1 and TGF-β1 (with concentrations 10 and 100 ng/mL) compared to WO stimulation.

The sera levels were in general higher in animals having a sequence homolog to the human PIIBNP splice variant as compared to humans. One explanation for this could be that the animals from which sera was drawn might be relatively young whereas the skeletal development or cartilage turnover in adult humans, as mentioned, is low. This hypothesis is further supported by high PIIBNP levels found in FBS, fetal horse serum and amniotic fluid in which the type II collagen formation is expected to originate from the chondrogenesis of the embryo [29]. Sera from species as sheep and chicken do not have a homolog PIIBNP sequence and had, as expected, levels close to the LLOD of the assay.

In regards to the *ex vivo* analysis, secretion of PIIBNP from bovine explants was evaluated twice with usage of cartilage from two different cows (results illustrated in Figure 2A,B). In both experiments there were found to be a significant increase of PIIBNP secretion in conditions stimulated by 100 ng/mL TGF-β1 already from the first week of culturing compared to the vehicle. The same tendency was found in explants stimulated by IGF-1 although it was not significant before weeks 2 and 3 for experiment A. For both BEX experiments it was found that the PIIBNP levels in WO and FGF-2 treated explants decreased from about 50 to 0–15 ng/mL in weeks 2 and 3. When explants were treated with a catabolic agent (O+T) the levels of Pro-C2 was significantly lowered compared to unstimulated conditions. Furthermore the evaluated PIIBNP levels had a tendency to decrease over time for explants stimulated by less than 10 ng/mL of IGF-1 or TGF-β1. Interestingly the size of the PIIBNP fragments detected in supernatant by western blot seemed to be approximately 150 kDA. This is equal to the size of a whole α-chain of type II collagen. It is uncertain why the fragment is not cleaved from the α-chain when secreted from BEX.

When Pro-C2 was evaluated in the *ex vivo* model of human explants the levels of PIIBNP were in general lower compared to those of BEX. This model is a bit more complicated to run, since there are fewer chondrocytes present in this model and they are more difficult to keep alive and responsive to stimuli. FBS (0.5%) was therefore added to the media in order to improve the viability and responsiveness in this model; this does, however, produce a high background of PIIBNP, since chondrocytes are very active during embryogenesis which results in high PIIBNP levels in FBS as seen in Table 2. In our assay it was possible to detect a significantly increased secretion of PIIBNP in response to stimulation of 100 ng/mL IGF-1 during the first week, whereas there was no detectable effect of either TGF-β1 of FGF-2. As the explants were apparently not responsive to any of the applied stimuli at week 2 or 3, we tried to decrease stimulation from 3 times a week to once a week, in case of overstimulation, but this did not improve the outcome.

## 4. Experimental Section

### 4.1. Immunogen Identification, Alignment and Selection

The sequence was selected by blasting the two isotypes of type II collagen and the last 7 amino acids QDVRQPG (*M*_W_ 798.85 g/mol) at the *N*-terminal end of the PIINP. The selected sequence is unique for PIIBNP. The sequence of interest differs at this part from isotype I with 3 amino acids as shown in Table 3. When aligning isotype II of bovine, rat and mouse, with human protype II collagen (Table 3) rat, rabbit, chicken and mouse form differs from the human one whereas the bovine isotype II and horse sequence are equal to the human version. From the table it is further seen that other species such as rat, mouse, and bovine also have two isotypes of the sequence.

**Table 3:** The targeted procollagen sequence of type II collagen aligned with the corresponding sequence in other species. The highlighted aminoacids indicate how the sequences differ from the targeted procollagen sequence.

Species	Sequence	NCBI Reference
Human Col2α1, isoform 2 (target)	QDVRQPG	NP_149162.2
Human Col2α1, isoform 1	QDV 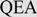 G	NP_001835.3
Bovine Col2α1, isoform 1	QDV 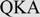 G	NP_001001135.2
Bovine Col2α1, isoform 2	QDVRQPG	NP_001106695.1
Mouse Col2α1, isoform 1	QD 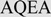 G	NP_112440.2
Mouse Col2α1, isoform 2	QD 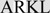 G	NP_001106987.2
Rat Col2α1	QD 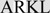 G	NP_037061.1
Rabbit Col2α1	QDV 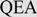 G	NP_001182600
Horse Col2α1	QDVRQPG	NP_001075233.1
Chicken Col2α1	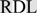 RQPG	NP_989757.1

### 4.2. Generation of the Monoclonal Antibody

A specific peptide with the sequence of QDVRQPG was synthesized and conjugated to maleimide-activated keyhole-limpet hemocyanin (Pierce, Beijing, China) as immunogen. 6, 4–6 week-old Balb/C mice, were injected subcutaneously with 50–60 μg of the immunogen emulsified with completed Freund’s adjuvant in equal volume (200 μL). Then two subcutaneous injections with 30 μg immunogen emulsified with incomplete Freund’s adjuvant were given two weeks apart, followed by four subcutaneous injections every-3-week. Mice bleedings were collected after third immunization. For each bleeding, affinity test for immunogen was carried out by adding a biotinylated (Bio) form of the screening peptide: Bio-QDVRQPG to streptavidin pre-coated micro-titer plate (Roche Diagnostics, Mannheim, Germany). The mouse with the highest titer was chosen for fusion. After one month rest, the selected mouse was boosted with 50 μg immunogen in 100 μL saline by intraperitoneal injection. Three days later, the mouse was terminated and the spleen was fused with SP2/0 cells. Hybridomas were cultured in the presences of hyposanthine/aminoptrein/thymidine (HAT) for one week. Then the media was replaced and the supernatants tested for reactivity against Bio-QDVRQPG on streptavidin coated micro-titer plates. Positive hybridomas were subcloned and the isotypes of the monoclonal antibodies were tested and only immunoglobulin G antibodies were selected. One monoclonal antibody, NB443-3-2-1, was chosen after thorough investigation based on affinity and specificity. Three synthetic peptides were used to test the specificity in inhibition ELISA: the selection peptide (QDVRQPG), the mouse/rat sequence for isoform I (QDARKLG), the human isotype I (QDVQEAG). No cross reactivity was observed for any of the sequences. All peptides applied in the study were synthesized by Scilight Biotechnology (Beijing, China).

### 4.3. Assay Development

A 96-well streptavidin plate (Roche Diagnostics) was coated with 1.25 ng of the biotinylated synthetic peptide, Bio-QDVRQPG, dissolved in assay buffer (100 mM phosphate buffered saline (PBS), 1% bovine serum albumin, 0.1% Tween-20, 0.36% Bronidox, 8% NaCl, adjusted to pH 7.4 at 20 °C) and incubated for 30 min at 20 °C. Twenty micro liters of the peptide calibrator or sample was added to appropriate wells, followed by 100 μL of 1:3000 diluted supernatant from the selected NB443-3-2-1 clone the plate was incubated for 2 h. Afterwards 100 μL EnVision diluted 1:30 was added and the plate was incubated for 1 h. Finally, 100 μL tetramethyl benzinidine (TMB) (Kem-En-Tec Nordic, Taastrup, Denmark) was added, and the plate was incubated for 15 min at 20 °C in the dark. All of the above incubation steps included shaking at 300 rpm. After each incubation step the plate was washed five times in washing buffer (20 mM Tris, 50 mM NaCl, pH 7.2). The TMB reaction was stopped by adding 100 μL of stopping solution (1% H_2_SO_4_) and measured at 450 nm with 650 nm as the reference.

Technical assay validation was done according to the international guide. The LLOD was computed as 3 standard deviation (SD) of the mean value of 20 zero standards. Intra-assay coefficient of variation (Intra-CV%, within a plate) and inter-assay coefficient of variation (Inter-CV%, between different plates) were calculated as the mean value of the variation of 5 samples analyzed 10 times in duplicate. Measurement range was defined as the range between lower limit of quantification (LLOQ), the lowest concentration of standard with a back-calculation of 100% ± 20%) and upper limit of quantification (LLOQ, back calculated Standard A mean value −3 SD). IC_50_ (half-maximal inhibition concentration) was calculated from the standard curve. Linearity dilution assessment and peptide spiking recovery assessment were performed to identify the matrix effect of serum sample.

### 4.4. Bovine and Human Cartilage Explants Culture

BEX was obtained from the femur condyle of animals aged 1–2 years from the local slaughterhouse (Harald Hansen, Slangerup, Denmark). The freshly slaughtered joint was cut open, and full thickness cartilage was obtained using a surgical scalpel and cut into homogenous explants of approximately 12 mg/piece by mean of a biopsy punch. HEX was obtained in collaboration with Gentofte hospital from 5 OA patients who had a knee replacement surgery. Explants were cut into homogenous explants in the same manner as BEX explants. There were 6 replicates for each treatment. The explants were placed in 96-well plates (Corning, Tewksbury, MA, USA) and rinsed 3 times in PBS (Invitrogen, Naerum, Denmark). The cartilage explants were incubated in serum-free Dulbecco’s modified Eagle’s medium (DMEM) supplemented with HAM’s F12-medium (DMEM:F12) (VWR, Soeborg, Denmark) + penicillin and streptavidin (Life Technologies Europe, Naerum, Denmark) at 37 °C, 5% CO_2_. In addition 0.5% fetal calf serum was added to the media of HEX. Following anabolic factors: fibroblastic growth factor (FGF)-2, insulin like growth factor (IGF)-1 and Transforming growth factor (TGF)-1β were then added at concentrations of 100, 10, 1 and 0.1 ng/mL (R&D systems, Abingdon, UK) to the media. The conditioned media was changed every 2–3 days. The HEX model was cultured for 6–21 days whereas the BEX model was cultured for 21 days and the media was stored at −20 °C until biochemical analysis. At termination of the experiment, chondrocyte viability was evaluated by the alamarBlue assay, according to the manufacture’s protocol (Trek Diagnostic Systems, East Grinstead, UK).

### 4.5. Western Blotting of BEX Supernatant

Supernatants were electrophoresed on a NuPAGE^®^Novex^®^ 4%–12% Bis–Tris gradient gel under reducing conditions using NuPAGE^®^ MES SDS running buffer (Life Technologies Europe BV, Naerum, Denmark). After transferring proteins from the polyacrylamide gel to the membrane by iBlot^®^ 7 min blotting system (Life Technologies Europe BV, Naerum, Denmark), the membrane was blocked with 20 mL blocking buffer (Tris-Buffered Saline (TBS) with 0.1%. Tween-20 and 5% skim milk powder from Fluka Analytical (Sigma–Aldrich Denmark ApS, Brøndby, Denmark) and shaken for 2 h. The membrane was incubated overnight at 4 °C with 1 µg/mL with NB443-3-2-1 as the primary antibody. After washing in TBST buffer (TBS with 0.1% Tween-20), the membrane was incubated with 1:5000 goat anti-mouse secondary antibody for 2 h. The membrane was washed with TBST buffer and enhanced chemiluminescence (ECL) western blotting substrate (GE healthcare, Brøndby, Denmark) was added for signals development. The bands were visualized through exposure to X-ray film.

### 4.6. Statistics

The ELISA standard curve was fitted by the 4-parameter method: *Y* = (*A* − *D*)/(1 + (*x*/*C*)^*B*) + *D*, where *R* > 0.9. Comparison between measurements of biomarkers in culture supernatants and differences between groups were assessed by one-way ANOVA followed by the non-parametric Mann Whitney test (α = 0.05) was used for comparison of subject groups. Error bars are shown as standard error of the mean (SEM).

## 5. Conclusions

It was enabled to measure cartilage formation by mean of a competitive ELISA assessing the propeptide PIIBNP, the beta splice variant of type II procollagen, which is expected to originate primarily from the remodeling of cartilage. Our further characterization of the developed Pro-C2 assay indicates a great potential for applying Pro-C2 as a tool for evaluating drug candidates of degenerative joint disease at least *ex vivo* or *in vitro*. To validate these indications more studies are needed and in particular human longitudinal studies are necessary for further evaluation of the potential of the Pro-C2 marker in degenerative joint diseases.

## Abbreviations

AGG-C1: A biomarker of an aggrecan specific neoepitope [19]
AGNX-II: A biomarker of an aggrecan specific neoepitope [18]
BEX: Bovine cartilage explants
Bio: Biotinylated
C2C: A biomarker of a collagen type II specific neoepitope
CIIM: A biomarker of a collagen type II specific neoepitope [13]
CIINE: A biomarker of a collagen type II specific neoepitope [16]
COMP: A biomarker for cartilage oligomeric matrix protein [20]
CTX-II: A biomarker of a type II collagen *C*-terminal telopeptide [14]
CV: Coefficient of variation
DJD: Degenerative joint disease
DMARD: Disease-modifying anti-rheumatic drugs
DMEM: Dulbecco’s modified Eagle’s medium
DMOARD: Disease modifying osteoarthritis drug
ECL: Enhanced chemiluminescence
ECM: Extra cellular matrix
FBS: Fetal bovine serum
FGF: Fibroblastic growth factor
HAT: Hyposanthine/aminoptrein/thymidine
HEX: Human cartilage explants
IGF: Insulin like growth factor
LLOD: Lower limit of detection
LLOQ: lower limit of quantification
MRI: Magnetic resonance imaging
OA: Osteoarthritis
PBS: Phosphate buffered saline
PIIANP: Isotype I of type II procollagen
PIIBNP: Isotype II of type II procollagen
PIICP: *C*-terminal propeptide of type II collagen
PIINP: *N*-terminal propeptide of type II collagen
RA: Rheumatoid arthritis
SD: Standard deviation
TBS: Tris-buffered Saline
TGF: Transforming growth factor
TIINE: A biomarker of a type II collagen inter-helical fragment [15]
TMB: Tetramethyl benzinidine
ULOQ: Upper limit of quantification
WO: without treatment
